# Rate-Distortion Function Upper Bounds for Gaussian Vectors and Their Applications in Coding AR Sources

**DOI:** 10.3390/e20060399

**Published:** 2018-05-23

**Authors:** Jesús Gutiérrez-Gutiérrez, Marta Zárraga-Rodríguez, Fernando M. Villar-Rosety, Xabier Insausti

**Affiliations:** Tecnun, University of Navarra, Paseo de Manuel Lardizábal 13, 20018 San Sebastián, Spain

**Keywords:** source coding, rate-distortion function (RDF), Gaussian vector, autoregressive (AR) source, discrete Fourier transform (DFT)

## Abstract

In this paper, we give upper bounds for the rate-distortion function (RDF) of any Gaussian vector, and we propose coding strategies to achieve such bounds. We use these strategies to reduce the computational complexity of coding Gaussian asymptotically wide sense stationary (AWSS) autoregressive (AR) sources. Furthermore, we also give sufficient conditions for AR processes to be AWSS.

## 1. Introduction

In 1956, Kolmogorov [1] gave a formula for the rate-distortion function (RDF) of Gaussian vectors and the RDF of Gaussian wide sense stationary (WSS) sources. Later, in 1970 Gray [2] obtained a formula for the RDF of Gaussian autoregressive (AR) sources.

In 1973, Pearl [3] gave an upper bound for the RDF of finite-length data blocks of Gaussian WSS sources, but he did not propose a coding strategy to achieve his bound for a given block length. In [4], we presented two tighter upper bounds for the RDF of finite-length data blocks of Gaussian WSS sources, and we proposed low-complexity coding strategies, based on the discrete Fourier transform (DFT), to achieve such bounds. Moreover, we proved that those two upper bounds tend to the RDF of the WSS source (computed by Kolmogorov in [1]) when the size of the data block grows.

In the present paper, we generalize the upper bounds and the two low-complexity coding strategies presented in [4] to any Gaussian vector. Therefore, in contrast to [4], here no assumption about the structure of the correlation matrix of the Gaussian vector has been made (observe that since the sources in [4] were WSS the correlation matrix of the Gaussian vectors there considered was Toeplitz). To obtain such generalization we start our analysis by first proving several new results on the DFT of random vectors. Although in [4] (Theorem 1) another new result on the DFT was presented, it cannot be used here, because such result and its proof rely on the power spectral density (PSD) of a WSS process and its properties.

The two low-complexity strategies here presented are applied in coding finite-length data blocks of Gaussian AR sources. Specifically, we prove that the rates (upper bounds) corresponding to these two strategies tend to the RDF of the AR source (computed by Gray in [2]) when the size of the data block grows and the AR source is asymptotically WSS (AWSS).

The definition of AWSS process was introduced by Gray in [5] (Chapter 6) and it is based on his concept of asymptotically equivalent sequences of matrices [6]. Sufficient conditions for AR processes to be AWSS can be found in [5] (Theorem 6.2) and [7] (Theorem 7). In this paper we present other sufficient conditions which make easier to check in practice whether an AR process is AWSS.

The paper is organized as follows. In Section 2 we obtain several new results on the DFT of random vectors which are used in Section 3. In Section 3 we give upper bounds for the RDF of Gaussian vectors, and we propose coding strategies to achieve such bounds. In Section 4 we apply the strategies proposed in Section 3 to reduce the computational complexity of coding Gaussian AWSS AR sources. In Section 5 we give sufficient conditions for AR processes to be AWSS. We finish the paper with a numerical example and conclusions.

## 2. Several New Results on the DFT of Random Vectors

We begin by introducing some notation. C denotes the set of (finite) complex numbers, i is the imaginary unit, Re and Im denote real and imaginary parts, respectively. * stands for conjugate transpose, ⊤ denotes transpose, and $\lambda_{k}\left(A\right)$, k∈{1,…,n}, are the eigenvalues of an n×n Hermitian matrix *A* arranged in decreasing order. *E* stands for expectation, and $V_{n}$ is the n×n Fourier unitary matrix, i.e.,

$${\left[V_{n}\right]}_{j,k}=\frac{1}{\sqrt{n}}e^{-\frac{2\pi (j-1)(k-1)}{n}i},\;j,k\in \left\{1,\ldots ,n\right\}.$$

If z∈C then $\hat{z}$ denotes the real (column) vector

z^=Re(z)Im(z).

If $z_{k}\in C$ for all k∈{1,…,n} then $z_{n:1}$ is the *n*-dimensional vector given by

$$z_{n:1}=\left(\begin{array}{c} z_{n}\\ z_{n-1}\\ z_{n-2}\\ \vdots \\ z_{1} \end{array}\right).$$

In this section, we give several new results on the DFT of random vectors in two theorems and one lemma.

**Theorem** **1.** Let $y_{n:1}$ be the DFT of an n-dimensional random vector $x_{n:1}$, that is, $y_{n:1}=V_{n}^{*}x_{n:1}$.

1. *If k∈{1,…,n} then*
  (1) $$\lambda_{n}\left(E\left(x_{n:1}x_{n:1}^{*}\right)\right)\leq E\left({\left|x_{k}\right|}^{2}\right)\leq \lambda_{1}\left(E\left(x_{n:1}x_{n:1}^{*}\right)\right)$$
  *and*
  (2) $$\lambda_{n}\left(E\left(x_{n:1}x_{n:1}^{*}\right)\right)\leq E\left({\left|y_{k}\right|}^{2}\right)\leq \lambda_{1}\left(E\left(x_{n:1}x_{n:1}^{*}\right)\right).$$
2. *If the random vector $x_{n:1}$ is real and $k\in \left\{1,\ldots ,n-1\right\}\setminus \left\{\frac{n}{2}\right\}$ then*
  (3) λn(Exn:1xn:1⊤)2≤ERe(yk)2≤λ1(Exn:1xn:1⊤)2,
  *and*
  (4) λn(Exn:1xn:1⊤)2≤EIm(yk)2≤λ1(Exn:1xn:1⊤)2.

**Proof.**  (1) We first prove that if $W_{n}$ is an n×n unitary matrix then

(5) $$\lambda_{n}\left(E\left(x_{n:1}x_{n:1}^{*}\right)\right)\leq {\left[W_{n}{\mathrm{diag}}_{1\leq j\leq n}\;\;\left(\lambda_{j}\left(E\left(x_{n:1}x_{n:1}^{*}\right)\right)\right)W_{n}^{*}\right]}_{n-k+1,n-k+1}\leq \lambda_{1}\left(E\left(x_{n:1}x_{n:1}^{*}\right)\right).$$

We have

(6) $$\begin{array}{cc} {\left[W_{n}{\mathrm{diag}}_{1\leq j\leq n}\;\;\left(\lambda_{j}\left(E\left(x_{n:1}x_{n:1}^{*}\right)\right)\right)W_{n}^{*}\right]}_{k_{1},k_{2}} & =\sum_{h=1}^{n}{\left[W_{n}\right]}_{k_{1},h}{\left[{\mathrm{diag}}_{1\leq j\leq n}\;\;\left(\lambda_{j}\left(E\left(x_{n:1}x_{n:1}^{*}\right)\right)\right)W_{n}^{*}\right]}_{h,k_{2}}\\ & =\sum_{h=1}^{n}{\left[W_{n}\right]}_{k_{1},h}\sum_{l=1}^{n}{\left[{\mathrm{diag}}_{1\leq j\leq n}\;\;\left(\lambda_{j}\left(E\left(x_{n:1}x_{n:1}^{*}\right)\right)\right)\right]}_{h,l}{\left[W_{n}^{*}\right]}_{l,k_{2}}\\ & =\sum_{h=1}^{n}{\left[W_{n}\right]}_{k_{1},h}\lambda_{h}\left(E\left(x_{n:1}x_{n:1}^{*}\right)\right)\bar{{\left[W_{n}\right]}_{k_{2},h}} \end{array}$$

for all $k_{1},k_{2}\in \left\{1,\ldots ,n\right\}$, and hence,

$${\left[W_{n}{\mathrm{diag}}_{1\leq j\leq n}\;\;\left(\lambda_{j}\left(E\left(x_{n:1}x_{n:1}^{*}\right)\right)\right)W_{n}^{*}\right]}_{n-k+1,n-k+1}=\sum_{h=1}^{n}\lambda_{h}\left(E\left(x_{n:1}x_{n:1}^{*}\right)\right){\left|{\left[W_{n}\right]}_{n-k+1,h}\right|}^{2}.$$

Consequently,

$$\begin{array}{cc} \lambda_{n}\left(E\left(x_{n:1}x_{n:1}^{*}\right)\right)\sum_{h=1}^{n}{\left|{\left[W_{n}\right]}_{n-k+1,h}\right|}^{2} & \leq {\left[W_{n}{\mathrm{diag}}_{1\leq j\leq n}\;\;\left(\lambda_{j}\left(E\left(x_{n:1}x_{n:1}^{*}\right)\right)\right)W_{n}^{*}\right]}_{n-k+1,n-k+1}\\ & \leq \lambda_{1}\left(E\left(x_{n:1}x_{n:1}^{*}\right)\right)\sum_{h=1}^{n}{\left|{\left[W_{n}\right]}_{n-k+1,h}\right|}^{2}, \end{array}$$

and applying

$$\sum_{h=1}^{n}{\left|{\left[W_{n}\right]}_{n-k+1,h}\right|}^{2}=\sum_{h=1}^{n}{\left[W_{n}\right]}_{n-k+1,h}{\left[W_{n}^{*}\right]}_{h,n-k+1}={\left[W_{n}W_{n}^{*}\right]}_{n-k+1,n-k+1}={\left[I_{n}\right]}_{n-k+1,n-k+1}=1,$$

where $I_{n}$ denotes the n×n identity matrix, we obtain Equation (5).

Let $E\left(x_{n:1}x_{n:1}^{*}\right)=U_{n}{\mathrm{diag}}_{1\leq j\leq n}\;\;\left(\lambda_{j}\left(E\left(x_{n:1}x_{n:1}^{*}\right)\right)\right)U_{n}^{-1}$ be a diagonalization of $E\left(x_{n:1}x_{n:1}^{*}\right)$ where the eigenvector matrix $U_{n}$ is unitary. As

$$E\left({\left|x_{k}\right|}^{2}\right)={\left[E\left(x_{n:1}x_{n:1}^{*}\right)\right]}_{n-k+1,n-k+1}={\left[U_{n}{\mathrm{diag}}_{1\leq j\leq n}\;\;\left(\lambda_{j}\left(E\left(x_{n:1}x_{n:1}^{*}\right)\right)\right)U_{n}^{*}\right]}_{n-k+1,n-k+1},$$

Equation (1) follows directly by taking $W_{n}=U_{n}$ in Equation (5).

Since

(7) $$\begin{array}{cc} E\left({\left|y_{k}\right|}^{2}\right) & ={\left[E\left(y_{n:1}y_{n:1}^{*}\right)\right]}_{n-k+1,n-k+1}\\ & ={\left[E\left(V_{n}^{*}x_{n:1}x_{n:1}^{*}{\left(V_{n}^{*}\right)}^{*}\right)\right]}_{n-k+1,n-k+1}\\ & ={\left[V_{n}^{*}E\left(x_{n:1}x_{n:1}^{*}\right){\left(V_{n}^{*}\right)}^{*}\right]}_{n-k+1,n-k+1}\\ & ={\left[V_{n}^{*}U_{n}{\mathrm{diag}}_{1\leq j\leq n}\;\;\left(\lambda_{j}\left(E\left(x_{n:1}x_{n:1}^{*}\right)\right)\right)U_{n}^{*}{\left(V_{n}^{*}\right)}^{*}\right]}_{n-k+1,n-k+1}\\ & ={\left[V_{n}^{*}U_{n}{\mathrm{diag}}_{1\leq j\leq n}\;\;\left(\lambda_{j}\left(E\left(x_{n:1}x_{n:1}^{*}\right)\right)\right){\left(V_{n}^{*}U_{n}\right)}^{*}\right]}_{n-k+1,n-k+1}, \end{array}$$

taking $W_{n}=V_{n}^{*}U_{n}$ in Equation (5) we obtain Equation (2).

(2) Applying [4] (Equation (10)) and taking $W_{n}=U_{n}$ in Equation (6) yields

ERe(yk)2=1n∑k1,k2=1ncos2π(1−k1)kncos2π(1−k2)knExn−k1+1xn−k2+1=1n∑k1,k2=1ncos2π(1−k1)kncos2π(1−k2)knExn:1xn:1⊤k1,k2=1n∑k1,k2=1ncos2π(1−k1)kncos2π(1−k2)knUndiag1≤j≤nλjExn:1xn:1⊤Un*k1,k2=1n∑k1,k2=1ncos2π(1−k1)kncos2π(1−k2)kn∑h=1n[Un]k1,hλhExn:1xn:1⊤[Un]k2,h¯=1n∑h=1nλhExn:1xn:1⊤∑k1=1ncos2π(1−k1)kn[Un]k1,h∑k2=1ncos2π(1−k2)kn[Un]k2,h¯=1n∑h=1nλhExn:1xn:1⊤∑l=1ncos2π(1−l)kn[Un]l,h2,

and therefore,

λnExn:1xn:1⊤1n∑h=1n∑l=1ncos2π(1−l)kn[Un]l,h2      ≤ERe(yk)2≤λ1Exn:1xn:1⊤1n∑h=1n∑l=1ncos2π(1−l)kn[Un]l,h2.

Analogously, it can be proved that

λnExn:1xn:1⊤1n∑h=1n∑l=1nsin2π(1−l)kn[Un]l,h2      ≤EIm(yk)2≤λ1Exn:1xn:1⊤1n∑h=1n∑l=1nsin2π(1−l)kn[Un]l,h2.

To finish the proof we only need to show that

(8) $$\frac{1}{n}\sum_{h=1}^{n}{\left|\sum_{l=1}^{n}\cos\frac{2\pi (1-l)k}{n}{\left[U_{n}\right]}_{l,h}\right|}^{2}=\frac{1}{n}\sum_{h=1}^{n}{\left|\sum_{l=1}^{n}\sin\frac{2\pi (1-l)k}{n}{\left[U_{n}\right]}_{l,h}\right|}^{2}=\frac{1}{2}.$$

If $b_{1},\ldots ,b_{n}$ are *n* real numbers then

(9) $$\begin{array}{cc} \frac{1}{n}\sum_{h=1}^{n}{\left|\sum_{l=1}^{n}b_{l}{\left[U_{n}\right]}_{l,h}\right|}^{2} & \;\;=\;\frac{1}{n}\sum_{h=1}^{n}\left(\sum_{k_{1}=1}^{n}b_{k_{1}}{\left[U_{n}\right]}_{k_{1},h}\right)\;\left(\sum_{k_{2}=1}^{n}b_{k_{2}}\bar{{\left[U_{n}\right]}_{k_{2},h}}\right)\;=\;\frac{1}{n}\sum_{k_{1},k_{2}=1}^{n}b_{k_{1}}b_{k_{2}}\sum_{h=1}^{n}{\left[U_{n}\right]}_{k_{1},h}{\left[U_{n}^{*}\right]}_{h,k_{2}}\\ & \;\;=\;\frac{1}{n}\sum_{k_{1},k_{2}=1}^{n}b_{k_{1}}b_{k_{2}}{\left[U_{n}U_{n}^{*}\right]}_{k_{1},k_{2}}\;=\;\frac{1}{n}\sum_{k_{1},k_{2}=1}^{n}b_{k_{1}}b_{k_{2}}{\left[I_{n}\right]}_{k_{1},k_{2}}\;=\;\frac{1}{n}\sum_{l=1}^{n}b_{l}^{2}, \end{array}$$

and thus,

$$\begin{array}{cc} \frac{1}{n}\sum_{h=1}^{n}{\left|\sum_{l=1}^{n}\sin\frac{2\pi (1-l)k}{n}{\left[U_{n}\right]}_{l,h}\right|}^{2} & \;\;=\;\frac{1}{n}\sum_{l=1}^{n}{\left(\sin\frac{2\pi (1-l)k}{n}\right)}^{2}\;=\;\frac{1}{n}\sum_{l=1}^{n}\left(1\;-\;{\left(\cos\frac{2\pi (1-l)k}{n}\right)}^{2}\right)\\ & \;\;=\;1\;-\;\frac{1}{n}\sum_{l=1}^{n}{\left(\cos\frac{2\pi (1-l)k}{n}\right)}^{2}\;=\;1\;-\;\frac{1}{n}\sum_{h=1}^{n}{\left|\sum_{l=1}^{n}\cos\frac{2\pi (1-l)k}{n}{\left[U_{n}\right]}_{l,h}\right|}^{2}\;\;. \end{array}$$

Equation (8) now follows directly from [4] (Equation (15)). ☐

**Lemma** **1.** *Let $y_{n:1}$ be the DFT of an n-dimensional random vector $x_{n:1}$. If k∈{1,…,n} then*

1. $E\left({\left|y_{k}\right|}^{2}\right)={\left[V_{n}^{*}E\left(x_{n:1}x_{n:1}^{*}\right)V_{n}\right]}_{n-k+1,n-k+1}$.
2. $E\left(y_{k}^{2}\right)={\left[V_{n}^{*}E\left(x_{n:1}x_{n:1}^{⊤}\right)\bar{V_{n}}\right]}_{n-k+1,n-k+1}$.
3. *EReykImyk=12ImEyk2.*
4. *ERe(yk)2=Eyk2+ReEyk22.*
5. *EIm(yk)2=Eyk2−ReEyk22.*

**Proof.**  (1) It is a direct consequence of Equation (7).

(2) We have

$$\begin{array}{cc} E\left(y_{k}^{2}\right) & ={\left[E\left(y_{n:1}y_{n:1}^{⊤}\right)\right]}_{n-k+1,n-k+1}={\left[E\left(V_{n}^{*}x_{n:1}x_{n:1}^{⊤}{\left(V_{n}^{*}\right)}^{⊤}\right)\right]}_{n-k+1,n-k+1}\\ & ={\left[E\left(V_{n}^{*}x_{n:1}x_{n:1}^{⊤}\bar{V_{n}}\right)\right]}_{n-k+1,n-k+1}={\left[V_{n}^{*}E\left(x_{n:1}x_{n:1}^{⊤}\right)\bar{V_{n}}\right]}_{n-k+1,n-k+1}. \end{array}$$

(3) Observe that

(10) Eyk2=ERe(yk)2−Im(yk)2+2Re(yk)Im(yk)i=ERe(yk)2−EIm(yk)2+2ERe(yk)Im(yk)i,

and hence,

ImEyk2=2ERe(yk)Im(yk).

(4) and (5) From Equation (10) we obtain

(11) ReEyk2=ERe(yk)2−EIm(yk)2.

Furthermore,

(12) Eyk2=ERe(yk)2+Im(yk)2=ERe(yk)2+EIm(yk)2.

(4) and (5) follow directly from Equations (11) and (12). ☐

**Theorem** **2.** *Let $y_{n:1}$ be the DFT of a real n-dimensional random vector $x_{n:1}$. If $k\in \left\{1,\ldots ,n-1\right\}\setminus \left\{\frac{n}{2}\right\}$ then*

$$\frac{\lambda_{n}\left(E\left(x_{n:1}x_{n:1}^{⊤}\right)\right)}{2}\leq \lambda_{2}\left(E\left(\hat{y_{k}}{\left(\hat{y_{k}}\right)}^{⊤}\right)\right)\leq \lambda_{1}\left(E\left(\hat{y_{k}}{\left(\hat{y_{k}}\right)}^{⊤}\right)\right)\leq \frac{\lambda_{1}\left(E\left(x_{n:1}x_{n:1}^{⊤}\right)\right)}{2}.$$

**Proof.**  Fix r∈{1,2} and consider a real unit eigenvector $v={\left(v_{1},v_{2}\right)}^{⊤}$ corresponding to $\lambda_{r}\left(E\left(\hat{y_{k}}{\left(\hat{y_{k}}\right)}^{⊤}\right)\right)$. We have

$$\lambda_{r}\left(E\left(\hat{y_{k}}{\left(\hat{y_{k}}\right)}^{⊤}\right)\right)=\lambda_{r}\left(E\left(\hat{y_{k}}{\left(\hat{y_{k}}\right)}^{⊤}\right)\right)v^{⊤}v=v^{⊤}\left(\lambda_{r}\left(E\left(\hat{y_{k}}{\left(\hat{y_{k}}\right)}^{⊤}\right)\right)v\right)=v^{⊤}E\left(\hat{y_{k}}{\left(\hat{y_{k}}\right)}^{⊤}\right)v.$$

From [4] (Equation (10)) we obtain

$$\begin{array}{c} \;\;\;\;E\left(\hat{y_{k}}{\left(\hat{y_{k}}\right)}^{⊤}\right)\\ =\frac{1}{n}\;\;\sum_{k_{1},k_{2}=1}^{n}\;\;\left(\begin{array}{cc} \;\cos\frac{2\pi (1\;-\;k_{1})k}{n}\cos\frac{2\pi (1\;-\;k_{2})k}{n}E\left(x_{n\;-\;k_{1}\;+\;1}x_{n\;-\;k_{2}\;+\;1}\right)\; & \;\cos\frac{2\pi (1\;-\;k_{1})k}{n}\sin\frac{2\pi (1\;-\;k_{2})k}{n}E\left(x_{n\;-\;k_{1}\;+\;1}x_{n\;-\;k_{2}\;+\;1}\right)\;\\ \sin\frac{2\pi (1\;-\;k_{1})k}{n}\cos\frac{2\pi (1\;-\;k_{2})k}{n}E\left(x_{n\;-\;k_{1}\;+\;1}x_{n\;-\;k_{2}\;+\;1}\right)\; & \;\sin\frac{2\pi (1\;-\;k_{1})k}{n}\sin\frac{2\pi (1\;-\;k_{2})k}{n}E\left(x_{n\;-\;k_{1}\;+\;1}x_{n\;-\;k_{2}\;+\;1}\right)\; \end{array}\right)\\ =\frac{1}{n}\sum_{k_{1},k_{2}=1}^{n}{\left[E\left(x_{n:1}x_{n:1}^{⊤}\right)\right]}_{k_{1},k_{2}}w_{k_{1}}w_{k_{2}}^{⊤} \end{array}$$

with

$$\begin{array}{c} w_{l}=\left(\begin{array}{c} \cos\frac{2\pi (1-l)k}{n}\\ \sin\frac{2\pi (1-l)k}{n} \end{array}\right),\;l\in \left\{1,\ldots ,n\right\}, \end{array}$$

and consequently,

$$\begin{array}{cc} \lambda_{r}\left(E\left(\hat{y_{k}}{\left(\hat{y_{k}}\right)}^{⊤}\right)\right) & =\frac{1}{n}\sum_{k_{1},k_{2}=1}^{n}{\left[E\left(x_{n:1}x_{n:1}^{⊤}\right)\right]}_{k_{1},k_{2}}v^{⊤}w_{k_{1}}w_{k_{2}}^{⊤}v\\ & =\frac{1}{n}\sum_{k_{1},k_{2}=1}^{n}\sum_{h=1}^{n}{\left[U_{n}\right]}_{k_{1},h}\lambda_{h}\left(E\left(x_{n:1}x_{n:1}^{⊤}\right)\right)\bar{{\left[U_{n}\right]}_{k_{2},h}}v^{⊤}w_{k_{1}}w_{k_{2}}^{⊤}v\\ & =\frac{1}{n}\;\sum_{k_{1},k_{2}=1}^{n}\;{\left(w_{k_{1}}^{⊤}v\right)}^{⊤}\;\sum_{h=1}^{n}{\left[U_{n}\right]}_{k_{1},h}\lambda_{h}\left(E\left(x_{n:1}x_{n:1}^{⊤}\right)\right)\bar{{\left[U_{n}\right]}_{k_{2},h}}w_{k_{2}}^{⊤}v\\ & =\frac{1}{n}\sum_{h=1}^{n}\lambda_{h}\left(E\left(x_{n:1}x_{n:1}^{⊤}\right)\right)\left(\sum_{k_{1}=1}^{n}w_{k_{1}}^{⊤}v{\left[U_{n}\right]}_{k_{1},h}\right)\left(\sum_{k_{2}=1}^{n}w_{k_{2}}^{⊤}v\bar{{\left[U_{n}\right]}_{k_{2},h}}\right)\\ & =\frac{1}{n}\sum_{h=1}^{n}\lambda_{h}\left(E\left(x_{n:1}x_{n:1}^{⊤}\right)\right){\left|\sum_{l=1}^{n}w_{l}^{⊤}v{\left[U_{n}\right]}_{l,h}\right|}^{2} \end{array}$$

with $E\left(x_{n:1}x_{n:1}^{⊤}\right)=U_{n}{\mathrm{diag}}_{1\leq j\leq n}\;\;\left(\lambda_{j}\left(E\left(x_{n:1}x_{n:1}^{⊤}\right)\right)\right)U_{n}^{-1}$ being a diagonalization of $E\left(x_{n:1}x_{n:1}^{⊤}\right)$ where the eigenvector matrix $U_{n}$ is unitary. Therefore,

$$\begin{array}{c} \lambda_{n}\left(\;E\left(x_{n:1}x_{n:1}^{⊤}\right)\;\right)\;\frac{1}{n}\sum_{h=1}^{n}{\left|\sum_{l=1}^{n}w_{l}^{⊤}v{\left[U_{n}\right]}_{l,h}\right|}^{2}\;\;\leq \;\lambda_{r}\left(\;E\left(\hat{y_{k}}{\left(\hat{y_{k}}\right)}^{⊤}\right)\;\right)\;\;\leq \;\lambda_{1}\left(\;E\left(x_{n:1}x_{n:1}^{⊤}\right)\;\right)\;\frac{1}{n}\sum_{h=1}^{n}{\left|\sum_{l=1}^{n}w_{l}^{⊤}v{\left[U_{n}\right]}_{l,h}\right|}^{2}\;\;. \end{array}$$

To finish the proof we only need to show that

$$\frac{1}{n}\sum_{h=1}^{n}{\left|\sum_{l=1}^{n}w_{l}^{⊤}v{\left[U_{n}\right]}_{l,h}\right|}^{2}=\frac{1}{2}.$$

Applying Equation (9) and [4] (Equations (14) and (15)) yields

1n∑h=1n∑l=1nwl⊤v[Un]l,h2=1n∑l=1nwl⊤v2=1n∑l=1ncos2π(1−l)knv1+sin2π(1−l)knv22=v121n∑l=1ncos2π(1−l)kn2+v221n∑l=1nsin2π(1−l)kn2+2v1v21n∑l=1ncos2π(1−l)knsin2π(1−l)kn=v121n∑l=1ncos2π(1−l)kn2+v222+v1v21n∑l=1nsin4π(1−l)kn=v121n∑l=1n1−sin2π(1−l)kn2+v222−v1v21n∑l=1nsin4π(l−1)kn=v121−1n∑l=1nsin2π(1−l)kn2+v222−v1v21n∑l=1nIme4π(l−1)kni=v122+v222−v1v21nIm∑l=1ne4π(l−1)kni=12v⊤v=12.

☐

## 3. RDF Upper Bounds for Real Gaussian Vectors

We first review the formula for the RDF of a real Gaussian vector given by Kolmogorov in [1].

**Theorem** **3.** *If $x_{n:1}$ is a real zero-mean Gaussian n-dimensional vector with positive definite correlation matrix, its RDF is given by*

$$R_{x_{n:1}}\left(D\right)=\frac{1}{n}\sum_{k=1}^{n}\max\left\{0,\frac{1}{2}\ln\frac{\lambda_{k}\left(E\left(x_{n:1}x_{n:1}^{⊤}\right)\right)}{\theta }\right\}\;\forall D\in \left(0,\frac{\mathrm{tr}\left(E\left(x_{n:1}x_{n:1}^{⊤}\right)\right)}{n}\right],$$

*where tr denotes trace and θ is a real number satisfying*

$$D=\frac{1}{n}\sum_{k=1}^{n}\min\left\{\theta ,\lambda_{k}\left(E\left(x_{n:1}x_{n:1}^{⊤}\right)\right)\right\}.$$

We recall that $R_{x_{n:1}}\left(D\right)$ can be thought of as the minimum rate (measured in nats) at which one must encode (compress) $x_{n:1}$ in order to be able to recover it with a mean square error (MSE) per dimension not larger than *D*, that is:

$$\frac{E\left({∥x_{n:1}-\tilde{x_{n:1}}∥}_{2}^{2}\right)}{n}\leq D,$$

where $\tilde{x_{n:1}}$ denotes the estimation of $x_{n:1}$ and ${∥\cdot ∥}_{2}$ is the spectral norm.

The following result provides an optimal coding strategy for $x_{n:1}$ in order to achieve $R_{x_{n:1}}\left(D\right)$ whenever $D\leq \lambda_{n}\left(E\left(x_{n:1}x_{n:1}^{⊤}\right)\right)$. Observe that if $D\leq \lambda_{n}\left(E\left(x_{n:1}x_{n:1}^{⊤}\right)\right)$ then

(13) $$R_{x_{n:1}}\left(D\right)=\frac{1}{2n}\sum_{k=1}^{n}\ln\frac{\lambda_{k}\left(E\left(x_{n:1}x_{n:1}^{⊤}\right)\right)}{D}=\frac{1}{2n}\ln\frac{\det\left(E\left(x_{n:1}x_{n:1}^{⊤}\right)\right)}{D^{n}}.$$

**Corollary** **1.** *Suppose that $x_{n:1}$ is as in Theorem 3. Let $E\left(x_{n:1}x_{n:1}^{⊤}\right)=U_{n}{\mathrm{diag}}_{1\leq k\leq n}\;\;\left(\lambda_{k}\left(E\left(x_{n:1}x_{n:1}^{⊤}\right)\right)\right)U_{n}^{-1}$ be a diagonalization of $E\left(x_{n:1}x_{n:1}^{⊤}\right)$ where the eigenvector matrix $U_{n}$ is real and orthogonal. If $D\in \left(0,\lambda_{n}\left(E\left(x_{n:1}x_{n:1}^{⊤}\right)\right)\right]$ then*

(14) $$R_{x_{n:1}}\left(D\right)=\frac{1}{n}\sum_{k=1}^{n}R_{z_{k}}\left(D\right)=\frac{1}{2n}\sum_{k=1}^{n}\ln\frac{E\left(z_{k}^{2}\right)}{D}$$

*with $z_{n:1}=U_{n}^{⊤}x_{n:1}$.*

**Proof.**  We encode $z_{1},\ldots ,z_{n}$ separately with $E\left({∥z_{k}-\tilde{z_{k}}∥}_{2}^{2}\right)\leq D$ for all k∈{1,…,n}. Let $\tilde{x_{n:1}}:=\;U_{n}\tilde{z_{n:1}}$, where

$$\tilde{z_{n:1}}:=\left(\begin{array}{c} \tilde{z_{n}}\\ \vdots \\ \tilde{z_{1}} \end{array}\right).$$

As $U_{n}^{⊤}$ is unitary (in fact, it is a real orthogonal matrix) and the spectral norm is unitarily invariant, we have

$$\begin{array}{cc} \frac{E\left({∥x_{n:1}-\tilde{x_{n:1}}∥}_{2}^{2}\right)}{n} & =\frac{E\left({∥U_{n}^{⊤}x_{n:1}-U_{n}^{⊤}\tilde{x_{n:1}}∥}_{2}^{2}\right)}{n}=\frac{E\left({∥z_{n:1}-\tilde{z_{n:1}}∥}_{2}^{2}\right)}{n}\\ & =\frac{E\left(\sum_{k=1}^{n}{\left(z_{k}-\tilde{z_{k}}\right)}^{2}\right)}{n}=\frac{\sum_{k=1}^{n}E\left({\left(z_{k}-\tilde{z_{k}}\right)}^{2}\right)}{n}=\frac{\sum_{k=1}^{n}E\left({∥z_{k}-\tilde{z_{k}}∥}_{2}^{2}\right)}{n}\leq D, \end{array}$$

and thus,

$$R_{x_{n:1}}\left(D\right)\leq \frac{1}{n}\sum_{k=1}^{n}R_{z_{k}}\left(D\right).$$

To finish the proof we show Equation (14). Since

$$E\left(z_{n:1}z_{n:1}^{⊤}\right)=E\left(U_{n}^{⊤}x_{n:1}x_{n:1}^{⊤}U_{n}\right)=U_{n}^{⊤}E\left(x_{n:1}x_{n:1}^{⊤}\right)U_{n}={\mathrm{diag}}_{1\leq k\leq n}\;\;\left(\lambda_{k}\left(E\left(x_{n:1}x_{n:1}^{⊤}\right)\right)\right),$$

we obtain

$$E\left(z_{k}^{2}\right)={\left[E\left(z_{n:1}z_{n:1}^{⊤}\right)\right]}_{n-k+1,n-k+1}=\lambda_{n-k+1}\left(E\left(x_{n:1}x_{n:1}^{⊤}\right)\right)\geq \lambda_{n}\left(E\left(x_{n:1}x_{n:1}^{⊤}\right)\right)\geq D>0.$$

Hence, applying Equation (13) yields

$$\begin{array}{cc} \frac{1}{n}\sum_{k=1}^{n}R_{z_{k}}\left(D\right) & =\frac{1}{n}\sum_{k=1}^{n}\frac{1}{2}\ln\frac{E\left(z_{k}^{2}\right)}{D}=\frac{1}{2n}\sum_{k=1}^{n}\ln\frac{\lambda_{n-k+1}\left(E\left(x_{n:1}x_{n:1}^{⊤}\right)\right)}{D}\\ & =\frac{1}{2n}\sum_{k=1}^{n}\ln\frac{\lambda_{k}\left(E\left(x_{n:1}x_{n:1}^{⊤}\right)\right)}{D}=R_{x_{n:1}}\left(D\right). \end{array}$$

☐

Corollary 1 shows that an optimal coding strategy for $x_{n:1}$ is to encode $z_{1},\ldots ,z_{n}$ separately.

We now give two coding strategies for $x_{n:1}$ based on the DFT whose computational complexity is lower than the computational complexity of the optimal coding strategy provided in Corollary 1.

**Theorem** **4.** *Let $x_{n:1}$ be as in Theorem 3. Suppose that $y_{n:1}$ is the DFT of $x_{n:1}$ and $D\in \left(0,\lambda_{n}\left(E\left(x_{n:1}x_{n:1}^{⊤}\right)\right)\right]$. Then*

(15) $$\begin{array}{cc} R_{x_{n:1}}\left(D\right) & \leq {\tilde{R}}_{x_{n:1}}\left(D\right)\leq {\overset{˘}{R}}_{x_{n:1}}\left(D\right)\leq \frac{1}{2n}\sum_{k=1}^{n}\ln\frac{E(|y_{k}{|}^{2})}{D} \end{array}$$

(16) $$\begin{array}{c} \leq R_{x_{n:1}}\left(D\right)+\frac{1}{2}\ln\left(1+\frac{{∥E\left(x_{n:1}x_{n:1}^{⊤}\right)-V_{n}{\mathrm{diag}}_{1\leq k\leq n}\;\;\left({\left[V_{n}^{*}E\left(x_{n:1}x_{n:1}^{⊤}\right)V_{n}\right]}_{k,k}\right)V_{n}^{*}∥}_{F}}{\sqrt{n}\;\lambda_{n}\left(E\left(x_{n:1}x_{n:1}^{⊤}\right)\right)}\right), \end{array}$$

*where ${∥\cdot ∥}_{F}$ is the Frobenius norm,*

$${\tilde{R}}_{x_{n:1}}\left(D\right):=\left\{\begin{array}{cc} \frac{R_{y_{\frac{n}{2}}}\left(D\right)+2\sum_{k=\frac{n}{2}+1}^{n-1}R_{\hat{y_{k}}}\left(\frac{D}{2}\right)+R_{y_{n}}\left(D\right)}{n} & if\;n\;is\;even,\;\\ \frac{2\sum_{k=\frac{n+1}{2}}^{n-1}R_{\hat{y_{k}}}\left(\frac{D}{2}\right)+R_{y_{n}}\left(D\right)}{n} & if\;n\;is\;odd, \end{array}\right.$$

*and*

R˘xn:1(D):=Ryn2D+∑k=n2+1n−1RReykD2+RImykD2+Ryn(D)nifniseven,∑k=n+12n−1RReykD2+RImykD2+Ryn(D)nifnisodd.

**Proof.**  Equations (15) and (16) were presented in [4] (Equations (16) and (20)) for the case where the correlation matrix $E\left(x_{n:1}x_{n:1}^{⊤}\right)$ is Toeplitz. They were proved by using a result on the DFT of random vectors with Toeplitz correlation matrix, namely, ref. [4] (Theorem 1). The proof of Theorem 4 is similar to the proof of [4] (Equations (16) and (20)) but using Theorem 1 instead of [4] (Theorem 1). Observe that in Theorems 1 and 4 no assumption about the structure of $E\left(x_{n:1}x_{n:1}^{⊤}\right)$ has been made. ☐

Theorem 4 shows that a coding strategy for $x_{n:1}$ is to encode $y_{\left\lceil \frac{n}{2}\right\rceil },\ldots ,y_{n}$ separately, where $\left\lceil \frac{n}{2}\right\rceil$ denotes the smallest integer higher than or equal to $\frac{n}{2}$. Theorem 4 also shows that another coding strategy for $x_{n:1}$ is to encode separately the real part and the imaginary part of $y_{k}$ instead of encoding $y_{k}$ when $k\in \left\{\left\lceil \frac{n}{2}\right\rceil ,\ldots ,n-1\right\}\setminus \left\{\frac{n}{2}\right\}$. The computational complexity of these two coding strategies based on the DFT is lower than the computational complexity of the optimal coding strategy provided in Corollary 1. Specifically, the complexity of computing the DFT ($y_{n:1}=V_{n}^{*}x_{n:1}$) is O(nlogn) whenever the fast Fourier transform (FFT) algorithm is used, while the complexity of computing $z_{n:1}=U_{n}^{⊤}x_{n:1}$ is $O(n^{2})$. Moreover, when the coding strategies based on the DFT are used, we do not need to compute a real orthogonal eigenvector matrix $U_{n}$ of $E\left(x_{n:1}x_{n:1}^{⊤}\right)$. It should also be mentioned that for these coding strategies based on the DFT the knowledge of $E\left(x_{n:1}x_{n:1}^{⊤}\right)$ is not even required, in fact, for them we only need to know $E\left(\hat{y_{k}}{\left(\hat{y_{k}}\right)}^{⊤}\right)$ with $k\in \{\left\lceil \frac{n}{2}\right\rceil ,\ldots ,n\}$.

The rates corresponding to the two coding strategies given in Theorem 4, ${\tilde{R}}_{x_{n:1}}\left(D\right)$ and ${\overset{˘}{R}}_{x_{n:1}}\left(D\right)$, can be written in terms of $E\left(x_{n:1}x_{n:1}^{⊤}\right)$ and $V_{n}$ by using Lemma 1 and the following lemma.

**Lemma** **2.** *Let $y_{n:1}$ and D be as in Theorem 4. Then*

1. *$R_{y_{k}}\left(D\right)=\frac{1}{2}\ln\frac{E\left(y_{k}^{2}\right)}{D}$ for all $k\in \left\{1,\ldots ,n\right\}\cap \left\{\frac{n}{2},n\right\}$.*
2. *Ryk^D2=14lnEReyk2EImyk2−EReykImyk2D22 for all $k\in \left\{1,\ldots ,n-1\right\}\setminus \left\{\frac{n}{2}\right\}$.*
3. *RReykD2=12lnEReyk2D2 for all $k\in \left\{1,\ldots ,n-1\right\}\setminus \left\{\frac{n}{2}\right\}$.*
4. *RImykD2=12lnEImyk2D2 for all $k\in \left\{1,\ldots ,n-1\right\}\setminus \left\{\frac{n}{2}\right\}$.*

**Proof.**  (1) Applying Equation (2) and [4] (Lemma 1) yields

$$0<D\leq \lambda_{n}\left(E\left(x_{n:1}x_{n:1}^{⊤}\right)\right)\leq E\left({\left|y_{k}\right|}^{2}\right)=E\left(y_{k}^{2}\right).$$

Assertion (1) now follows directly from Equation (13).

(2) Applying Theorem 2 we have

$$0<\frac{D}{2}\leq \frac{\lambda_{n}\left(E\left(x_{n:1}x_{n:1}^{⊤}\right)\right)}{2}\leq \lambda_{2}\left(E\left(\hat{y_{k}}{\left(\hat{y_{k}}\right)}^{⊤}\right)\right).$$

Consequently, from Equation (13) we obtain

Ryk^D2=14lndetEyk^yk^⊤D22=14lndetEReyk2EReykImykEImykReykEImyk2D22.

Assertions (3) and (4) Applying Equations (3) and (4) yields

0<D2≤λnExn:1xn:1⊤2≤EReyk2.

and

0<D2≤λnExn:1xn:1⊤2≤EImyk2.

Assertions (3) and (4) now follow directly from Equation (13). ☐

We end this section with a result that is a direct consequence of Lemma 2. This result shows when the rates corresponding to the two coding strategies given in Theorem 4, ${\tilde{R}}_{x_{n:1}}\left(D\right)$ and ${\overset{˘}{R}}_{x_{n:1}}\left(D\right)$, are equal.

**Lemma** **3.** *Let $x_{n:1}$, $y_{n:1}$, and D be as in Theorem 4. Then the two following assertions are equivalent:*

1. *${\tilde{R}}_{x_{n:1}}\left(D\right)={\overset{˘}{R}}_{x_{n:1}}\left(D\right)$.*
2. *EReykImyk=0 for all $k\in \left\{\left\lceil \frac{n}{2}\right\rceil ,\ldots ,n-1\right\}\setminus \left\{\frac{n}{2}\right\}$.*

**Proof.**  Fix $k\in \left\{\left\lceil \frac{n}{2}\right\rceil ,\ldots ,n-1\right\}\setminus \left\{\frac{n}{2}\right\}$. From Lemma 2 we have

2Ryk^D2=12lnEReyk2EImyk2−EReykImyk2D22≤12lnEReyk2EImyk2D22=12lnEReyk2D2+12lnEImyk2D2=RReykD2+RImykD2.

☐

## 4. Low-Complexity Coding Strategies for Gaussian AWSS AR Sources

We begin by introducing some notation. The symbols N, Z, and R denote the set of positive integers, integers, and (finite) real numbers, respectively. If f:R→C is continuous and 2π-periodic, we denote by $T_{n}\left(f\right)$ the n×n Toeplitz matrix given by

$${\left[T_{n}\left(f\right)\right]}_{j,k}=t_{j-k},$$

where ${\left\{t_{k}\right\}}_{k\in Z}$ is the sequence of Fourier coefficients of *f*, i.e.,

$$t_{k}=\frac{1}{2\pi }\int_{0}^{2\pi }f\left(\omega \right)e^{-k\omega i}d\omega \;\forall k\in Z.$$

If $A_{n}$ and $B_{n}$ are n×n matrices for all n∈N, we write $\left\{A_{n}\right\}∼\left\{B_{n}\right\}$ if the sequences $\left\{A_{n}\right\}$ and $\left\{B_{n}\right\}$ are asymptotically equivalent, that is, $\left\{∥A_{n}{∥}_{2}\right\}$ and $\left\{∥B_{n}{∥}_{2}\right\}$ are bounded and $\lim_{n\to \infty }\frac{∥A_{n}-B_{n}{∥}_{F}}{\sqrt{n}}=0$ (see [5] (Section 2.3) and [6]).

We now review the definitions of AWSS processes and AR processes.

**Definition** **1.** A random process $\left\{x_{n}\right\}$ is said to be AWSS if it has constant mean (i.e., $E\left(x_{j}\right)=E\left(x_{k}\right)$ for all j,k∈N) and there exists a continuous 2π-periodic function f:R→C such that $\left\{E\left(x_{n:1}x_{n:1}^{*}\right)\right\}∼\left\{T_{n}\left(f\right)\right\}$. The function f is called (asymptotic) PSD of $\left\{x_{n}\right\}$.

**Definition** **2.** *A real zero-mean random process $\left\{x_{n}\right\}$ is said to be AR if*

$$x_{n}=w_{n}-\sum_{k=1}^{n-1}a_{-k}x_{n-k}\;\forall n\in N,$$

*or equivalently,*

(17) $$\sum_{k=0}^{n-1}a_{-k}x_{n-k}=w_{n}\;\forall n\in N,$$

*where $a_{0}=1$, $a_{-k}\in R$ for all k∈N, and $\left\{w_{n}\right\}$ is a real zero-mean random process satisfying that $E\left(w_{j}w_{k}\right)=\delta_{j,k}\sigma^{2}$ for all j,k∈N with $\sigma^{2}>0$ and $\delta_{j,k}$ being the Kronecker delta (i.e., $\delta_{j,k}=1$ if j=k, and it is zero otherwise).*

*The AR process $\left\{x_{n}\right\}$ in Equation (17) is of finite order if there exists p∈N such that $a_{-k}=0$ for all k>p. In this case, $\left\{x_{n}\right\}$ is called an AR (p) process.*

The following theorem shows that if $x_{n:1}$ is a large enough data block of a Gaussian AWSS AR source, the rate does not increase whenever we encode it using the two coding strategies based on the DFT presented in Section 3, instead of encoding $x_{n:1}$ using an eigenvector matrix of its correlation matrix.

**Theorem** **5.** *Let $\left\{x_{n}\right\}$ be as in Definition 2. Suppose that ${\left\{a_{k}\right\}}_{k\in Z}$, with $a_{k}=0$ for all k∈N, is the sequence of Fourier coefficients of a function a:R→C which is continuous and 2π-periodic. Then*

1. *$\inf_{n\in N}\lambda_{n}\left(E\left(x_{n:1}x_{n:1}^{⊤}\right)\right)\geq \frac{\sigma^{2}}{\max_{\omega \in [0,2\pi ]}{\left|a\left(\omega \right)\right|}^{2}}>0$.*
2. *Consider $D\in \left(0,\inf_{n\in N}\lambda_{n}\left(E\left(x_{n:1}x_{n:1}^{⊤}\right)\right)\right]$.*
  (*a*) *If $\left\{x_{n}\right\}$ is Gaussian,*
    (18) $$\frac{1}{2}\ln\frac{\sigma^{2}}{D}\;=\;R_{x_{n:1}}\left(\;D\;\right)\;\leq \;{\tilde{R}}_{x_{n:1}}\left(\;D\;\right)\;\leq \;{\overset{˘}{R}}_{x_{n:1}}\left(\;D\;\right)\;\leq \;K_{1}\left(n,D\;\right)\;\leq \;K_{2}\left(n,D\;\right)\;\leq \;K_{3}\left(n,D\;\right)\;\forall n\in N,$$
    *where $K_{1}\left(n,D\right)$ is given by Equation (16), and $K_{2}\left(n,D\right)$ and $K_{3}\left(n,D\right)$ are obtained by replacing $\lambda_{n}\left(E\left(x_{n:1}x_{n:1}^{⊤}\right)\right)$ in Equation (16) by $\inf_{n\in N}\lambda_{n}\left(E\left(x_{n:1}x_{n:1}^{⊤}\right)\right)$ and $\frac{\sigma^{2}}{\max_{\omega \in [0,2\pi ]}{\left|a\left(\omega \right)\right|}^{2}}$, respectively.*
  (*b*) *If $\left\{x_{n}\right\}$ is Gaussian and AWSS,*
    (19) $$\lim_{n\to \infty }R_{x_{n:1}}\left(D\right)=\lim_{n\to \infty }{\tilde{R}}_{x_{n:1}}\left(D\right)=\lim_{n\to \infty }{\overset{˘}{R}}_{x_{n:1}}\left(D\right)=\lim_{n\to \infty }K_{3}\left(n,D\right).$$

**Proof.** (1) Equation (17) can be rewritten as

$$T_{n}\left(a\right)x_{n:1}=w_{n:1}\;\forall n\in N.$$

Consequently,

$$T_{n}\left(a\right)E\left(x_{n:1}x_{n:1}^{⊤}\right){\left(T_{n}\left(a\right)\right)}^{⊤}=E\left(T_{n}\left(a\right)x_{n:1}{\left(T_{n}\left(a\right)x_{n:1}\right)}^{⊤}\right)=E\left(w_{n:1}w_{n:1}^{⊤}\right)=\sigma^{2}I_{n}\;\forall n\in N.$$

As $\det(T_{n}\left(a\right))=1$, $T_{n}\left(a\right)$ is invertible, and therefore,

(20) $$\begin{array}{cc} E\left(x_{n:1}x_{n:1}^{⊤}\right) & =\sigma^{2}{\left(T_{n}\left(a\right)\right)}^{-1}{\left({\left(T_{n}\left(a\right)\right)}^{⊤}\right)}^{-1}=\sigma^{2}{\left({\left(T_{n}\left(a\right)\right)}^{⊤}T_{n}\left(a\right)\right)}^{-1}=\sigma^{2}{\left({\left(T_{n}\left(a\right)\right)}^{*}T_{n}\left(a\right)\right)}^{-1}\\ & =\sigma^{2}{\left(N_{n}{\mathrm{diag}}_{1\leq k\leq n}\left({\left(\sigma_{k}\left(T_{n}\left(a\right)\right)\right)}^{2}\right)N_{n}^{*}\right)}^{-1}=N_{n}{\mathrm{diag}}_{1\leq k\leq n}\left(\frac{\sigma^{2}}{{\left(\sigma_{k}\left(T_{n}\left(a\right)\right)\right)}^{2}}\right)N_{n}^{*} \end{array}$$

for all n∈N, where $T_{n}\left(a\right)=M_{n}{\mathrm{diag}}_{1\leq k\leq n}\left(\sigma_{k}\left(T_{n}\left(a\right)\right)\right)N_{n}^{*}$ is a singular value decomposition of $T_{n}\left(a\right)$. Thus, applying [8] (Theorem 4.3) yields

$$\lambda_{n}\left(E\left(x_{n:1}x_{n:1}^{⊤}\right)\right)=\frac{\sigma^{2}}{{\left(\sigma_{1}\left(T_{n}\left(a\right)\right)\right)}^{2}}\geq \frac{\sigma^{2}}{\max_{\omega \in [0,2\pi ]}{\left|a\left(\omega \right)\right|}^{2}}>0\;\forall n\in N.$$

(2a) From Equation (13) we have

$$\begin{array}{cc} R_{x_{n:1}}\left(D\right) & =\frac{1}{2n}\ln\frac{\det\left(E\left(x_{n:1}x_{n:1}^{⊤}\right)\right)}{D^{n}}=\frac{1}{2n}\ln\frac{\det\left(\sigma^{2}{\left(T_{n}\left(a\right)\right)}^{-1}{\left({\left(T_{n}\left(a\right)\right)}^{⊤}\right)}^{-1}\right)}{D^{n}}\\ & =\frac{1}{2n}\ln\frac{{\left(\sigma^{2}\right)}^{n}}{D^{n}\det\left(T_{n}\left(a\right)\right)\det\left({\left(T_{n}\left(a\right)\right)}^{⊤}\right)}=\frac{1}{2n}\ln\frac{{\left(\sigma^{2}\right)}^{n}}{D^{n}}=\frac{1}{2}\ln\frac{\sigma^{2}}{D}\;\forall n\in N. \end{array}$$

Assertion (2a) now follows from Theorem 4 and Assertion (1).

(2b) From Assertion (2a) we only need to show that

(21) $$\lim_{n\to \infty }\frac{{∥E\left(x_{n:1}x_{n:1}^{⊤}\right)-V_{n}{\mathrm{diag}}_{1\leq k\leq n}\;\;\left({\left[V_{n}^{*}E\left(x_{n:1}x_{n:1}^{⊤}\right)V_{n}\right]}_{k,k}\right)V_{n}^{*}∥}_{F}}{\sqrt{n}}=0.$$

As the Frobenius norm is unitarily invariant we obtain

$$\begin{array}{cc} 0 & \;\leq \;\frac{{∥E\left(x_{n:1}x_{n:1}^{⊤}\right)-V_{n}{\mathrm{diag}}_{1\leq k\leq n}\;\;\left({\left[V_{n}^{*}E\left(x_{n:1}x_{n:1}^{⊤}\right)V_{n}\right]}_{k,k}\right)V_{n}^{*}∥}_{F}}{\sqrt{n}}\\ & \;\leq \;\frac{{∥E\;\left(x_{n:1}x_{n:1}^{⊤}\right)\;-\;T_{n}\left(f\right)∥}_{F}}{\sqrt{n}}\;+\;\frac{{∥T_{n}\left(f\right)\;-\;{\hat{C}}_{n}\left(f\right)∥}_{F}}{\sqrt{n}}\;+\;\frac{{∥V_{n}{\mathrm{diag}}_{1\leq k\leq n}\;\;\left(\;{\left[V_{n}^{*}E\;\left(x_{n:1}x_{n:1}^{⊤}\right)V_{n}\right]}_{k,k}\;\right)\;V_{n}^{*}\;-\;{\hat{C}}_{n}\left(f\right)∥}_{F}}{\sqrt{n}}\\ & \;=\;\frac{{∥E\;\left(x_{n:1}x_{n:1}^{⊤}\right)\;-\;T_{n}\left(f\right)∥}_{F}}{\sqrt{n}}\;+\;\frac{{∥T_{n}\left(f\right)\;-\;{\hat{C}}_{n}\left(f\right)∥}_{F}}{\sqrt{n}}\;+\;\frac{{∥V_{n}{\mathrm{diag}}_{1\leq k\leq n}\;\;\left(\;{\left[V_{n}^{*}\;\left(E\;\left(x_{n:1}x_{n:1}^{⊤}\right)\;-\;T_{n}\left(f\right)\right)\;V_{n}\right]}_{k,k}\;\right)\;\;V_{n}^{*}∥}_{F}}{\sqrt{n}}\\ & \;=\;\frac{{∥E\;\left(x_{n:1}x_{n:1}^{⊤}\right)\;-\;T_{n}\left(f\right)∥}_{F}}{\sqrt{n}}\;+\;\frac{{∥T_{n}\left(f\right)\;-\;{\hat{C}}_{n}\left(f\right)∥}_{F}}{\sqrt{n}}\;+\;\frac{{∥{\mathrm{diag}}_{1\leq k\leq n}\;\;\left(\;{\left[V_{n}^{*}\left(E\;\left(x_{n:1}x_{n:1}^{⊤}\right)-T_{n}\left(f\right)\right)V_{n}\right]}_{k,k}\;\right)∥}_{F}}{\sqrt{n}}\\ & \;\leq \;\frac{{∥E\;\left(x_{n:1}x_{n:1}^{⊤}\right)\;-\;T_{n}\left(f\right)∥}_{F}}{\sqrt{n}}\;+\;\frac{{∥T_{n}\left(f\right)\;-\;{\hat{C}}_{n}\left(f\right)∥}_{F}}{\sqrt{n}}\;+\;\frac{{∥V_{n}^{*}\left(E\;\left(x_{n:1}x_{n:1}^{⊤}\right)-T_{n}\left(f\right)\right)V_{n}∥}_{F}}{\sqrt{n}}\\ & \;=\;2\frac{{∥E\;\left(x_{n:1}x_{n:1}^{⊤}\right)\;-\;T_{n}\left(f\right)∥}_{F}}{\sqrt{n}}\;+\;\frac{{∥T_{n}\left(f\right)\;-\;{\hat{C}}_{n}\left(f\right)∥}_{F}}{\sqrt{n}}\;, \end{array}$$

where *f* is (asymptotic) PSD of $\left\{x_{n}\right\}$ and ${\hat{C}}_{n}\left(f\right)=V_{n}{\mathrm{diag}}_{1\leq k\leq n}\left({\left[V_{n}^{*}T_{n}\left(f\right)V_{n}\right]}_{k,k}\right)V_{n}^{*}$. Assertion (2b) now follows from $\left\{E\left(x_{n:1}x_{n:1}^{⊤}\right)\right\}∼\left\{T_{n}\left(f\right)\right\}$ and [9] (Lemma 4.2). ☐

If $\sum_{k=-\infty }^{0}\left|a_{k}\right|<\infty$, there always exists such function *a* and it is given by $a\left(\omega \right)=\sum_{k=-\infty }^{0}a_{k}e^{k\omega i}$ for all ω∈R (see, e.g., [8] (Appendix B)). In particular, if $\left\{x_{n}\right\}$ is an AR (p) process, $a\left(\omega \right)=\sum_{k=-p}^{0}a_{k}e^{k\omega i}$ for all ω∈R.

## 5. Sufficient Conditions for AR Processes to be AWSS

In the following two results we give sufficient conditions for AR processes to be AWSS.

**Theorem** **6.** *Let $\left\{x_{n}\right\}$ be as in Definition 2. Suppose that ${\left\{a_{k}\right\}}_{k\in Z}$, with $a_{k}=0$ for all k∈N, is the sequence of Fourier coefficients of a function a:R→C which is continuous and 2π-periodic. Then the following assertions are equivalent:*

1. *$\left\{x_{n}\right\}$ is AWSS.*
2. *$\left\{{∥E\left(x_{n:1}x_{n:1}^{⊤}\right)∥}_{2}\right\}$ is bounded.*
3. *$\left\{T_{n}\left(a\right)\right\}$ is stable (that is, $\left\{∥{\left(T_{n}\left(a\right)\right)}^{-1}{∥}_{2}\right\}$ is bounded).*
4. *a(ω)≠0 for all ω∈R and $\left\{x_{n}\right\}$ is AWSS with (asymptotic) PSD $\frac{\sigma^{2}}{{\left|a\right|}^{2}}$.*

**Proof.**  (1)⇒(2) This is a direct consequence of the definition of AWSS process, i.e., of Definition 1.

(2)⇔(3) From Equation (20) we have

$${∥E\left(x_{n:1}x_{n:1}^{⊤}\right)∥}_{2}=\frac{\sigma^{2}}{{\left(\sigma_{n}\left(T_{n}\left(a\right)\right)\right)}^{2}}=\sigma^{2}{∥N_{n}{\mathrm{diag}}_{1\leq k\leq n}\left(\frac{1}{\sigma_{k}\left(T_{n}\left(a\right)\right)}\right)M_{n}^{*}∥}_{2}^{2}=\sigma^{2}{∥{\left(T_{n}\left(a\right)\right)}^{-1}∥}_{2}^{2}$$

for all n∈N.

(3)⇒(4) It is well known that if f:R→C is continuous and 2π-periodic, and $\left\{T_{n}\left(f\right)\right\}$ is stable then f(ω)≠0 for all ω∈R. Hence, a(ω)≠0 for all ω∈R.

Applying [8] (Lemma 4.2.1) yields $\left\{{\left(T_{n}\left(a\right)\right)}^{⊤}\right\}=\left\{{\left(T_{n}\left(a\right)\right)}^{*}\right\}=\left\{T_{n}\left(\bar{a}\right)\right\}$. Consequently, from [7] (Theorem 3) we obtain

$$\left\{{\left(T_{n}\left(a\right)\right)}^{⊤}T_{n}\left(a\right)\right\}=\left\{T_{n}\left(\bar{a}\right)T_{n}\left(a\right)\right\}∼\left\{T_{n}\left(\bar{a}a\right)\right\}=\left\{T_{n}\left({\left|a\right|}^{2}\right)\right\}.$$

Observe that the sequence

$$\left\{{∥{\left({\left(T_{n}\left(a\right)\right)}^{⊤}T_{n}\left(a\right)\right)}^{-1}∥}_{2}\right\}=\left\{{∥\frac{1}{\sigma^{2}}E\left(x_{n:1}x_{n:1}^{⊤}\right)∥}_{2}\right\}=\left\{\frac{1}{\sigma^{2}}{∥E\left(x_{n:1}x_{n:1}^{⊤}\right)∥}_{2}\right\}$$

is bounded. As the function ${\left|a\right|}^{2}$ is real, applying [8] (Theorem 4.4) we have that $T_{n}\left({\left|a\right|}^{2}\right)$ is Hermitian and $0<\min_{\omega \in [0,2\pi ]}{\left|a\left(\omega \right)\right|}^{2}\leq \lambda_{n}\left(T_{n}\left({\left|a\right|}^{2}\right)\right)$ for all n∈N, and therefore,

$${∥{\left(T_{n}\left({\left|a\right|}^{2}\right)\right)}^{-1}∥}_{2}=\frac{1}{\lambda_{n}\left(T_{n}\left({\left|a\right|}^{2}\right)\right)}\leq \frac{1}{\min_{\omega \in [0,2\pi ]}{\left|a\left(\omega \right)\right|}^{2}}\;\forall n\in N.$$

Thus, from [5] (Theorem 1.4) we obtain

$$\left\{\frac{1}{\sigma^{2}}E\left(x_{n:1}x_{n:1}^{⊤}\right)\right\}=\left\{{\left({\left(T_{n}\left(a\right)\right)}^{⊤}T_{n}\left(a\right)\right)}^{-1}\right\}∼\left\{{\left(T_{n}\left({\left|a\right|}^{2}\right)\right)}^{-1}\right\}.$$

Hence, applying [10] (Theorem 4.2) and [5] (Theorem 1.2) yields

$$\left\{\frac{1}{\sigma^{2}}E\left(x_{n:1}x_{n:1}^{⊤}\right)\right\}∼\left\{T_{n}\left(\frac{1}{{\left|a\right|}^{2}}\right)\right\}.$$

Consequently, from [8] (Lemma 3.1.3) and [8] (Lemma 4.2.3) we have

$$\left\{E\left(x_{n:1}x_{n:1}^{⊤}\right)\right\}∼\left\{\sigma^{2}T_{n}\left(\frac{1}{{\left|a\right|}^{2}}\right)\right\}=\left\{T_{n}\left(\frac{\sigma^{2}}{{\left|a\right|}^{2}}\right)\right\}.$$

(4)⇒(1) It is obvious.

**Corollary** **2.** *Let $\left\{x_{n}\right\}$ be as in Definition 2 with $\sum_{k=-\infty }^{0}\left|a_{k}\right|<\infty$. If $\sum_{k=0}^{\infty }a_{-k}z^{k}\neq 0$ for all |z|≤1 then $\left\{x_{n}\right\}$ is AWSS.*

**Proof.**  It is well known that if a sequence of complex numbers ${\left\{t_{k}\right\}}_{k\in Z}$ satisfies that $\sum_{k=-\infty }^{\infty }\left|t_{k}\right|<\infty$ and that $\sum_{k=-\infty }^{\infty }t_{k}z^{k}\neq 0$ for all |z|≤1 then $\left\{T_{n}\left(f\right)\right\}$ is stable with $f\left(\omega \right)=\sum_{k=-\infty }^{\infty }t_{k}e^{k\omega i}$ for all ω∈R. Therefore, $\left\{T_{n}\left(b\right)\right\}$ is stable with $b\left(\omega \right)=\sum_{k=0}^{\infty }a_{-k}e^{k\omega i}$ for all ω∈R. Thus,

$$\left\{{∥{\left(T_{n}\left(a\right)\right)}^{-1}∥}_{2}\right\}=\left\{{∥{\left({\left(T_{n}\left(a\right)\right)}^{-1}\right)}^{⊤}∥}_{2}\right\}=\left\{{∥{\left({\left(T_{n}\left(a\right)\right)}^{⊤}\right)}^{-1}∥}_{2}\right\}=\left\{{∥{\left(T_{n}\left(b\right)\right)}^{-1}∥}_{2}\right\}$$

is bounded with $a\left(\omega \right)=\sum_{k=-\infty }^{0}a_{k}e^{k\omega i}$ for all ω∈R. As $\left\{T_{n}\left(a\right)\right\}$ is stable, from Theorem 6 we conclude that $\left\{x_{n}\right\}$ is AWSS. ☐

## 6. Numerical Example and Conclusions

### 6.1. Example

**Figure 1:**
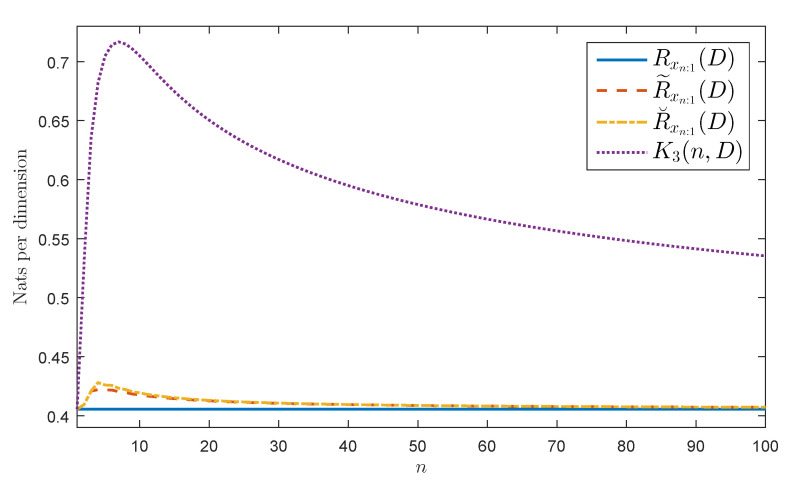
Considered rates for a Gaussian AWSS AR(1) source.

Let $\left\{x_{n}\right\}$ be as in Definition 2 with $a_{-k}=0$ for all k>1. Observe that $\frac{\sigma^{2}}{\max_{\omega \in [0,2\pi ]}{\left|a\left(\omega \right)\right|}^{2}}=\frac{\sigma^{2}}{\left(1+\right|a_{-1}{\left|\right)}^{2}}$. If $|a_{-1}|<1$ from Corollary 2 we obtain that the AR(1) process $\left\{x_{n}\right\}$ is AWSS. Figure 1 shows $R_{x_{n:1}}\left(D\right)$, ${\tilde{R}}_{x_{n:1}}\left(D\right)$, and ${\overset{˘}{R}}_{x_{n:1}}\left(D\right)$ by assuming that $\left\{x_{n}\right\}$ is Gaussian, $a_{-1}=-\frac{1}{2}$, $\sigma^{2}=1$, $D=\frac{\sigma^{2}}{\left(1+\right|a_{-1}{\left|\right)}^{2}}=\frac{4}{9}$, and n≤100. Figure 1 also shows the highest upper bound of $R_{x_{n:1}}\left(D\right)$ presented in Theorem 5, namely, $K_{3}\left(n,D\right)$. Observe that the figure bears evidence of the equalities and inequalities given in Equations (18) and (19).

### 6.2. Conclusions

The computational complexity of coding finite-length data blocks of Gaussian sources can be reduced by using any of the two low-complexity coding strategies here presented instead of the optimal coding strategy. Moreover, the rate does not increase if we use those strategies instead of the optimal one whenever the Gaussian source is AWSS and AR, and the considered data block is large enough.
